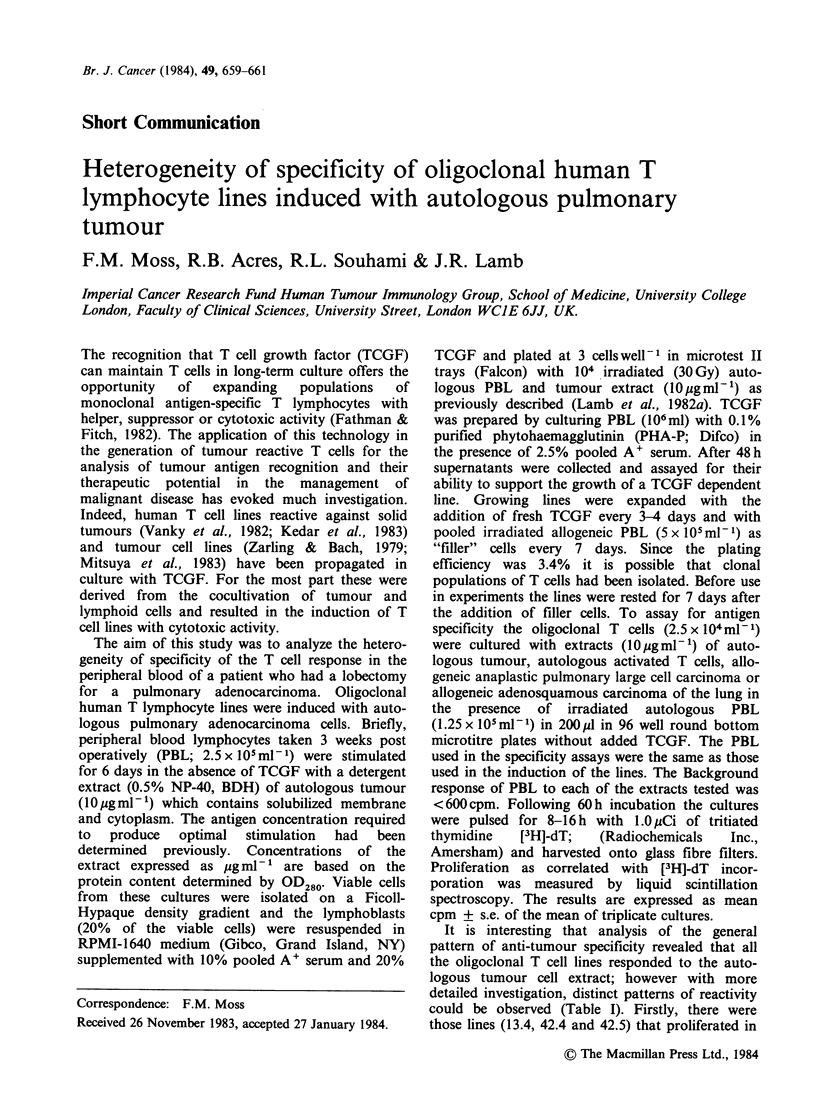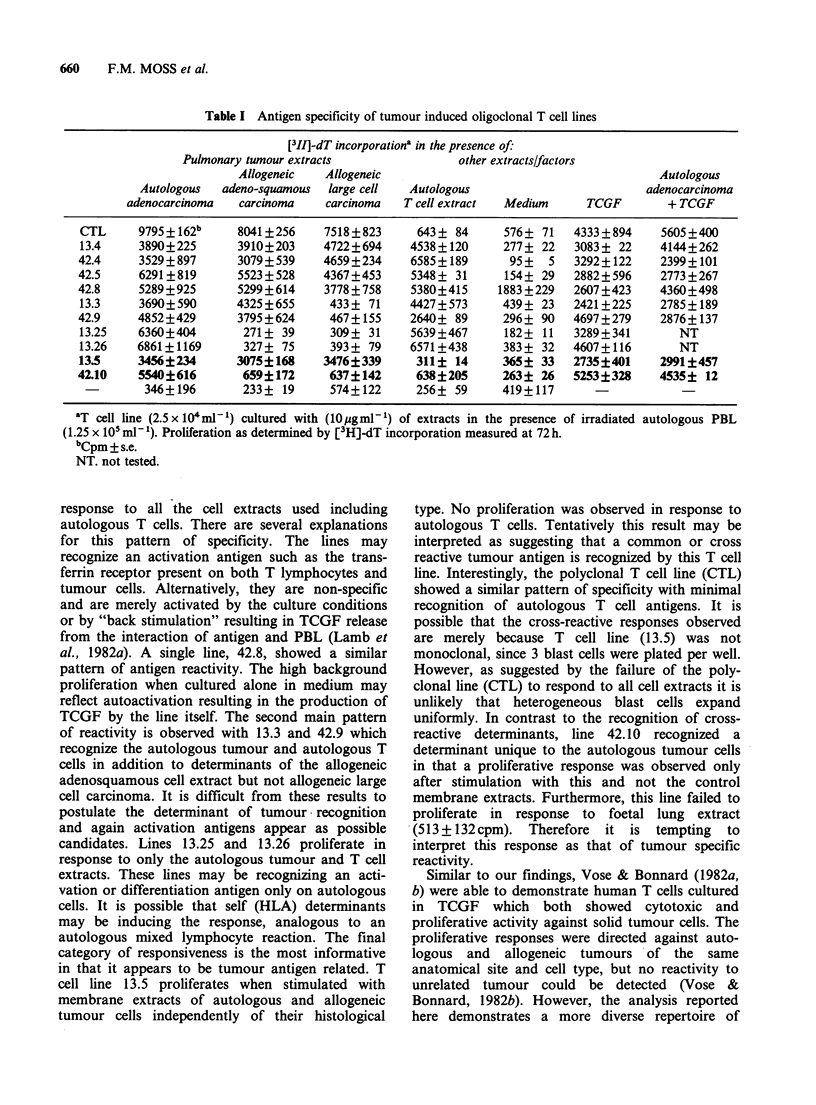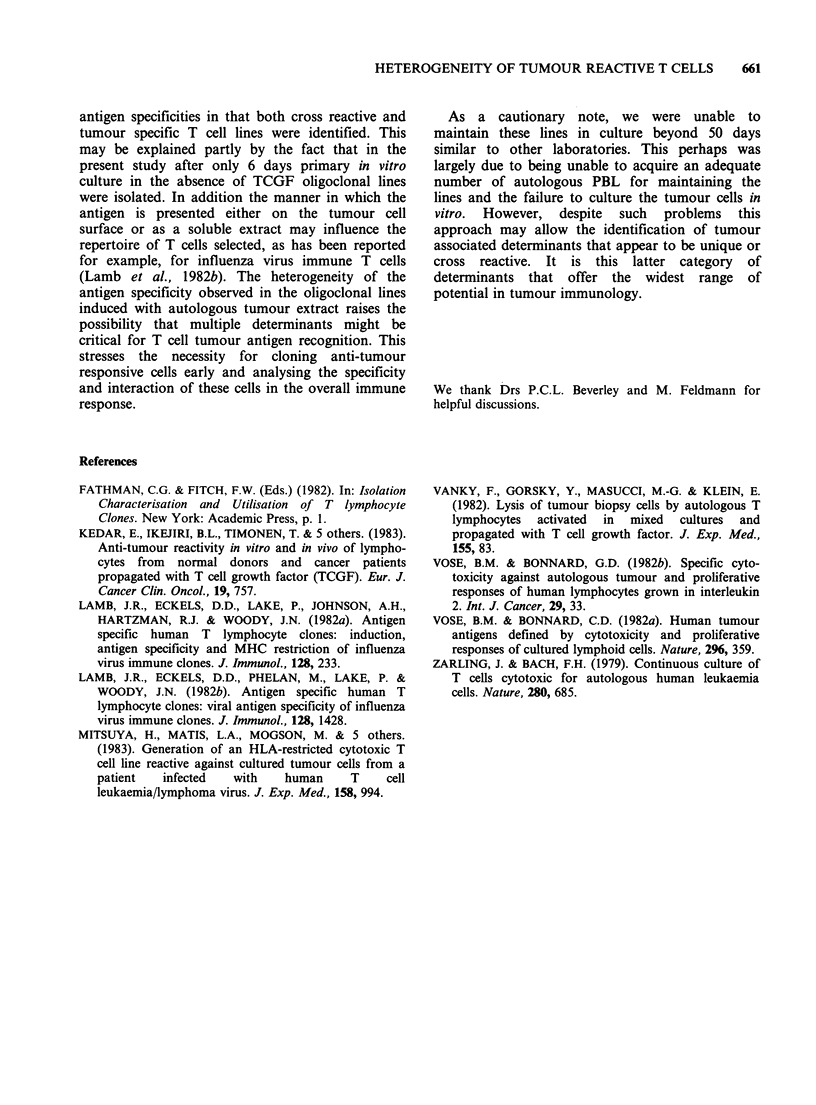# Heterogeneity of specificity of oligoclonal human T lymphocyte lines induced with autologous pulmonary tumour.

**DOI:** 10.1038/bjc.1984.102

**Published:** 1984-05

**Authors:** F. M. Moss, R. B. Acres, R. L. Souhami, J. R. Lamb


*Br. J. Cancer* (1984), **49**, 659–661

**Short Communication**

### Heterogeneity of specificity of oligoclonal human T lymphocyte lines induced with autologous pulmonary tumour

F.M. Moss, R.B. Acres, R.L. Souhami & J.R. Lamb

*Imperial Cancer Research Fund Human Tumour Immunology Group, School of Medicine, University College London, Faculty of Clinical Sciences, University Street, London WC1E 6JJ, UK.*

The recognition that T cell growth factor (TCGF) can maintain T cells in long-term culture offers the opportunity of expanding populations of monoclonal antigen-specific T lymphocytes with helper, suppressor or cytotoxic activity (Fathman & Fitch, 1982). The application of this technology in the generation of tumour reactive T cells for the analysis of tumour antigen recognition and their therapeutic potential in the management of malignant disease has evoked much investigation. Indeed, human T cell lines reactive against solid tumours (Vanky *et al.*, 1982; Kedar *et al.*, 1983) and tumour cell lines (Zarling & Bach, 1979; Mitsuya *et al.*, 1983) have been propagated in culture with TCGF. For the most part these were derived from the cocultivation of tumour and lymphoid cells and resulted in the induction of T cell lines with cytotoxic activity.

The aim of this study was to analyze the heterogeneity of specificity of the T cell response in the peripheral blood of a patient who had a lobectomy for a pulmonary adenocarcinoma. Oligoclonal human T lymphocyte lines were induced with autologous pulmonary adenocarcinoma cells. Briefly, peripheral blood lymphocytes taken 3 weeks post operatively (PBL; $2.5 \times 10^5 \text{ml}^{-1}$) were stimulated for 6 days in the absence of TCGF with a detergent extract (0.5% NP-40, BDH) of autologous tumour ($10 \mu\text{g ml}^{-1}$) which contains solubilized membrane and cytoplasm. The antigen concentration required to produce optimal stimulation had been determined previously. Concentrations of the extract expressed as $\mu\text{g ml}^{-1}$ are based on the protein content determined by $OD_{280}$. Viable cells from these cultures were isolated on a Ficoll-Hypaque density gradient and the lymphoblasts (20% of the viable cells) were resuspended in RPMI-1640 medium (Gibco, Grand Island, NY) supplemented with 10% pooled $A^+$ serum and 20% TCGF and plated at 3 cells $\text{well}^{-1}$ in microtest II trays (Falcon) with $10^4$ irradiated (30 Gy) autologous PBL and tumour extract ($10 \mu\text{g ml}^{-1}$) as previously described (Lamb *et al.*, 1982*a*). TCGF was prepared by culturing PBL ($10^6$ ml) with 0.1% purified phytohaemagglutinin (PHA-P; Difco) in the presence of 2.5% pooled $A^+$ serum. After 48 h supernatants were collected and assayed for their ability to support the growth of a TCGF dependent line. Growing lines were expanded with the addition of fresh TCGF every 3–4 days and with pooled irradiated allogeneic PBL ($5 \times 10^5 \text{ml}^{-1}$) as "filler" cells every 7 days. Since the plating efficiency was 3.4% it is possible that clonal populations of T cells had been isolated. Before use in experiments the lines were rested for 7 days after the addition of filler cells. To assay for antigen specificity the oligoclonal T cells ($2.5 \times 10^4 \text{ml}^{-1}$) were cultured with extracts ($10 \mu\text{g ml}^{-1}$) of autologous tumour, autologous activated T cells, allogeneic anaplastic pulmonary large cell carcinoma or allogeneic adenosquamous carcinoma of the lung in the presence of irradiated autologous PBL ($1.25 \times 10^5 \text{ml}^{-1}$) in 200 $\mu$l in 96 well round bottom microtitre plates without added TCGF. The PBL used in the specificity assays were the same as those used in the induction of the lines. The Background response of PBL to each of the extracts tested was $<600$ cpm. Following 60 h incubation the cultures were pulsed for 8–16 h with 1.0 $\mu$Ci of tritiated thymidine [$^3$H]-dT; (Radiochemicals Inc., Amersham) and harvested onto glass fibre filters. Proliferation as correlated with [$^3$H]-dT incorporation was measured by liquid scintillation spectroscopy. The results are expressed as mean cpm $\pm$ s.e. of the mean of triplicate cultures.

It is interesting that analysis of the general pattern of anti-tumour specificity revealed that all the oligoclonal T cell lines responded to the autologous tumour cell extract; however with more detailed investigation, distinct patterns of reactivity could be observed (Table I). Firstly, there were those lines (13.4, 42.4 and 42.5) that proliferated in

Correspondence: F.M. Moss

Received 26 November 1983, accepted 27 January 1984.

© The Macmillan Press Ltd., 1984

**Table I** Antigen specificity of tumour induced oligoclonal T cell lines

| | *[$^3$II]-dT incorporation*[a] *in the presence of:* *Pulmonary tumour extracts* | | | *other extracts/factors* | | | |
|---|---|---|---|---|---|---|---|
| | *Autologous adenocarcinoma* | *Allogeneic adeno-squamous carcinoma* | *Allogeneic large cell carcinoma* | *Autologous T cell extract* | *Medium* | *TCGF* | *Autologous adenocarcinoma + TCGF* |
| CTL | 9795±162[b] | 8041±256 | 7518±823 | 643± 84 | 576± 71 | 4333±894 | 5605±400 |
| 13.4 | 3890±225 | 3910±203 | 4722±694 | 4538±120 | 277± 22 | 3083± 22 | 4144±262 |
| 42.4 | 3529±897 | 3079±539 | 4659±234 | 6585±189 | 95± 5 | 3292±122 | 2399±101 |
| 42.5 | 6291±819 | 5523±528 | 4367±453 | 5348± 31 | 154± 29 | 2882±596 | 2773±267 |
| 42.8 | 5289±925 | 5299±614 | 3778±758 | 5380±415 | 1883±229 | 2607±423 | 4360±498 |
| 13.3 | 3690±590 | 4325±655 | 433± 71 | 4427±573 | 439± 23 | 2421±225 | 2785±189 |
| 42.9 | 4852±429 | 3795±624 | 467±155 | 2640± 89 | 296± 90 | 4697±279 | 2876±137 |
| 13.25 | 6360±404 | 271± 39 | 309± 31 | 5639±467 | 182± 11 | 3289±341 | NT |
| 13.26 | 6861±1169 | 327± 75 | 393± 79 | 6571±438 | 383± 32 | 4607±116 | NT |
| **13.5** | **3456±234** | **3075±168** | **3476±339** | **311± 14** | **365± 33** | **2735±401** | **2991±457** |
| **42.10** | **5540±616** | **659±172** | **637±142** | **638±205** | **263± 26** | **5253±328** | **4535± 12** |
| — | 346±196 | 233± 19 | 574±122 | 256± 59 | 419±117 | — | — |

[a]T cell line ($2.5 \times 10^4 ml^{-1}$) cultured with ($10 \mu g ml^{-1}$) of extracts in the presence of irradiated autologous PBL ($1.25 \times 10^5 ml^{-1}$). Proliferation as determined by [$^3$H]-dT incorporation measured at 72 h.

[b]Cpm ± s.e.

NT. not tested.

response to all the cell extracts used including autologous T cells. There are several explanations for this pattern of specificity. The lines may recognize an activation antigen such as the transferrin receptor present on both T lymphocytes and tumour cells. Alternatively, they are non-specific and are merely activated by the culture conditions or by "back stimulation" resulting in TCGF release from the interaction of antigen and PBL (Lamb *et al.*, 1982*a*). A single line, 42.8, showed a similar pattern of antigen reactivity. The high background proliferation when cultured alone in medium may reflect autoactivation resulting in the production of TCGF by the line itself. The second main pattern of reactivity is observed with 13.3 and 42.9 which recognize the autologous tumour and autologous T cells in addition to determinants of the allogeneic adenosquamous cell extract but not allogeneic large cell carcinoma. It is difficult from these results to postulate the determinant of tumour recognition and again activation antigens appear as possible candidates. Lines 13.25 and 13.26 proliferate in response to only the autologous tumour and T cell extracts. These lines may be recognizing an activation or differentiation antigen only on autologous cells. It is possible that self (HLA) determinants may be inducing the response, analogous to an autologous mixed lymphocyte reaction. The final category of responsiveness is the most informative in that it appears to be tumour antigen related. T cell line 13.5 proliferates when stimulated with membrane extracts of autologous and allogeneic tumour cells independently of their histological type. No proliferation was observed in response to autologous T cells. Tentatively this result may be interpreted as suggesting that a common or cross reactive tumour antigen is recognized by this T cell line. Interestingly, the polyclonal T cell line (CTL) showed a similar pattern of specificity with minimal recognition of autologous T cell antigens. It is possible that the cross-reactive responses observed are merely because T cell line (13.5) was not monoclonal, since 3 blast cells were plated per well. However, as suggested by the failure of the polyclonal line (CTL) to respond to all cell extracts it is unlikely that heterogeneous blast cells expand uniformly. In contrast to the recognition of cross-reactive determinants, line 42.10 recognized a determinant unique to the autologous tumour cells in that a proliferative response was observed only after stimulation with this and not the control membrane extracts. Furthermore, this line failed to proliferate in response to foetal lung extract (513±132 cpm). Therefore it is tempting to interpret this response as that of tumour specific reactivity.

Similar to our findings, Vose & Bonnard (1982*a*, *b*) were able to demonstrate human T cells cultured in TCGF which both showed cytotoxic and proliferative activity against solid tumour cells. The proliferative responses were directed against autologous and allogeneic tumours of the same anatomical site and cell type, but no reactivity to unrelated tumour could be detected (Vose & Bonnard, 1982*b*). However, the analysis reported here demonstrates a more diverse repertoire of antigen specificities in that both cross reactive and tumour specific T cell lines were identified. This may be explained partly by the fact that in the present study after only 6 days primary *in vitro* culture in the absence of TCGF oligoclonal lines were isolated. In addition the manner in which the antigen is presented either on the tumour cell surface or as a soluble extract may influence the repertoire of T cells selected, as has been reported for example, for influenza virus immune T cells (Lamb *et al.,* 1982*b*). The heterogeneity of the antigen specificity observed in the oligoclonal lines induced with autologous tumour extract raises the possibility that multiple determinants might be critical for T cell tumour antigen recognition. This stresses the necessity for cloning anti-tumour responsive cells early and analysing the specificity and interaction of these cells in the overall immune response.

As a cautionary note, we were unable to maintain these lines in culture beyond 50 days similar to other laboratories. This perhaps was largely due to being unable to acquire an adequate number of autologous PBL for maintaining the lines and the failure to culture the tumour cells *in vitro*. However, despite such problems this approach may allow the identification of tumour associated determinants that appear to be unique or cross reactive. It is this latter category of determinants that offer the widest range of potential in tumour immunology.

We thank Drs P.C.L. Beverley and M. Feldmann for helpful discussions.

### References

FATHMAN, C.G. & FITCH, F.W. (Eds.) (1982). In: *Isolation Characterisation and Utilisation of T lymphocyte Clones*. New York: Academic Press, p. 1.

KEDAR, E., IKEJIRI, B.L., TIMONEN, T. & 5 others. (1983). Anti-tumour reactivity *in vitro* and *in vivo* of lymphocytes from normal donors and cancer patients propagated with T cell growth factor (TCGF). *Eur. J. Cancer Clin. Oncol.*, **19**, 757.

LAMB, J.R., ECKELS, D.D., LAKE, P., JOHNSON, A.H., HARTZMAN, R.J. & WOODY, J.N. (1982*a*). Antigen specific human T lymphocyte clones: induction, antigen specificity and MHC restriction of influenza virus immune clones. *J. Immunol.*, **128**, 233.

LAMB, J.R., ECKELS, D.D., PHELAN, M., LAKE, P. & WOODY, J.N. (1982*b*). Antigen specific human T lymphocyte clones: viral antigen specificity of influenza virus immune clones. *J. Immunol.*, **128**, 1428.

MITSUYA, H., MATIS, L.A., MOGSON, M. & 5 others. (1983). Generation of an HLA-restricted cytotoxic T cell line reactive against cultured tumour cells from a patient infected with human T cell leukaemia/lymphoma virus. *J. Exp. Med.*, **158**, 994.

VANKY, F., GORSKY, Y., MASUCCI, M.-G. & KLEIN, E. (1982). Lysis of tumour biopsy cells by autologous T lymphocytes activated in mixed cultures and propagated with T cell growth factor. *J. Exp. Med.*, **155**, 83.

VOSE, B.M. & BONNARD, G.D. (1982*b*). Specific cytotoxicity against autologous tumour and proliferative responses of human lymphocytes grown in interleukin 2. *Int. J. Cancer*, **29**, 33.

VOSE, B.M. & BONNARD, C.D. (1982*a*). Human tumour antigens defined by cytotoxicity and proliferative responses of cultured lymphoid cells. *Nature*, **296**, 359.

ZARLING, J. & BACH, F.H. (1979). Continuous culture of T cells cytotoxic for autologous human leukaemia cells. *Nature*, **280**, 685.